# Unlocking Triticeae genomics to sustainably feed the future

**DOI:** 10.1093/pcp/pct163

**Published:** 2013-11-06

**Authors:** Keiichi Mochida, Kazuo Shinozaki

**Affiliations:** ^1^Biomass Research Platform Team, Biomass Engineering Program Cooperation Division, RIKEN Center for Sustainable Resource Science, 1-7-22 Suehiro-cho, Tsurumi-ku, Yokohama, Kanagawa, 230-0045 Japan; ^2^Kihara Institute for Biological Research, Yokohama City University, 641-12 Maioka-cho, Totsuka-ku, Yokohama, Kanagawa, 230-0045 Japan

**Keywords:** Barley, *Brachypodium*, Crop improvement, Next-generation sequencing, Triticeae, Wheat

## Abstract

The tribe Triticeae includes the major crops wheat and barley. Within the last few years, the whole genomes of four Triticeae species—barley, wheat, Tausch’s goatgrass (*Aegilops tauschii*) and wild einkorn wheat (*Triticum urartu*)—have been sequenced. The availability of these genomic resources for Triticeae plants and innovative analytical applications using next-generation sequencing technologies are helping to revitalize our approaches in genetic work and to accelerate improvement of the Triticeae crops. Comparative genomics and integration of genomic resources from Triticeae plants and the model grass *Brachypodium distachyon* are aiding the discovery of new genes and functional analyses of genes in Triticeae crops. Innovative approaches and tools such as analysis of next-generation populations, evolutionary genomics and systems approaches with mathematical modeling are new strategies that will help us discover alleles for adaptive traits to future agronomic environments. In this review, we provide an update on genomic tools for use with Triticeae plants and *Brachypodium* and describe emerging approaches toward crop improvements in Triticeae.

## Introduction

Ensuring global food security is an urgent challenge for our society. The world’s population is estimated to rise to 9 billion by 2050 (http://esa.un.org/unpd/wpp/index.htm), and global crop production will need to be increased in order to feed this population (Alexandratos and Bruinsma 2012). At the same time, the world’s agricultural systems are facing the risk of production shortfalls due to various abiotic and biotic stresses from global climate change (Wheeler and von Braun 2013). In order to develop crops that can better withstand climate change, we need to connect diverse and interdisciplinary scientific and technological advancements in crop breeding.

During the past decade, remarkable innovations in analytical platforms for omics-based research and development have provided crucial resources to promote research in model and applied plant species. A combinatorial approach using multiple omics platforms and integrating their outcomes has been an effective strategy to understand molecular systems that are critical to improving plant productivity (Mochida and Shinozaki 2010). Recent breathtaking advances in sequencing technologies have revitalized approaches in genomics and created various analytical applications (Mochida and Shinozaki 2011, Plocik and Graveley 2013).

Next-generation sequencing (NGS) technologies have accelerated whole-genome sequencing projects, sometimes in combination with the conventional Sanger sequencing methods. For instance, Phytozome, a joint project of the Department of Energy’s Joint Genome Institute and the Center for Integrative Genomics, has provided access to 41 sequenced and annotated plant genomes as of the latest release, v. 9.1 (http://www.phytozome.net/) (Goodstein et al. 2012). The latest release from Ensembl Plants, Release 20, includes information on genome sequence and associated annotations for 26 plant species, including the recently sequenced barley and wheat (http://plants.ensembl.org/index.html).

Whole-genome re-sequencing is a feasible NGS application to catalog genomic variations in natural, mapping and mutant populations, to find the association between genetics and biological consequences (Arai-Kichise et al. 2011, Ashelford et al. 2011, James et al. 2013, Miyao et al. 2012, Uchida et al. 2011). Genome-guided RNA-seq is an effective approach to identify transcription units and splice isoforms together with their expression profiles in genome-sequenced organisms (Filichkin et al. 2010, Loraine et al. 2013, Szczesniak et al. 2013). De novo RNA-seq has become a popular method to acquire initial information about transcribed sequences and gene expression profiles without a reference genome sequence, and there are many examples in various non-model organisms, including plants of particular biological and industrial interest (Fu et al. 2011, Su et al. 2011, Bai et al. 2013, Gil-Amado and Gomez-Jimenez 2013, Postnikova et al. 2013, Ramilowski et al. 2013, Tsai et al. 2013). The cDNA information reconstructed from de novo RNA-seq data sets has been useful as a proxy for the genic regions of the genome in that species (Hirsch and Buell 2013). Analyses based on high-throughput sequencing have rapidly become a significant approach in genome-wide biology for crop improvements. Herein, we focused on staple crops, wheat and barley, of the tribe Triticeae in the subfamily Pooideae. Bread wheat (*Triticum aestivum* L. subsp. *aestivum*) is one of the most important crops in the world, accounting for 20% of calories consumed by humans. Barley (*Hordeum vulgare* L.) is also a globally important crop that is utilized for animal feed, in the production of malt and as a human food source. In 2011, a special issue on barley was published in *Plant and Cell Physiology* to highlight the resources related to and achievements in Triticeae genomics at that time (Saisho and Takeda 2011). These available genomic resources in Triticeae have dramatically changed over the last 2 years, which provides us with an opportunity to design new combinations of tools to promote Triticeae genomics and to accelerate the process of gene discovery in genome-oriented breeding.

In this review, we provide an overview of recent advancements focused in Triticeae genomics. We first describe recent progress in genomic resources and their applications in Triticeae species, including wheat and barley. We then highlight the current status of genomic resources in the emerging model grass of the Pooideae subfamily, *Brachypodium distachyon* (L.) P. Beauv, and discuss its roles as a model plant for Triticeae species. Finally, we review several pioneering studies dealing with the association between genetic diversity and traits that are adaptive to the environment, with possible applications for future Triticeae genomics and molecular breeding. Throughout this review, we showcase recent genome-based efforts and available resources that are useful in Triticeae genomics.

## Genomic resources of Triticeae crops

About 10,000 years ago, in the Fertile Crescent in western Asia, humans made the transition from the hunter–gatherer way of life to a sedentary agrarian lifestyle. Domestication of einkorn wheat, emmer wheat and barley marked the beginning of agriculture in that area (Dubcovsky and Dvorak 2007). Barley (*H. vulgare*; 2*n* = 2*x* = 14; HH) was domesticated there from its wild relative, *H. vulgare* subsp. *spontaneum* (K.Koch) Thell. Domesticated einkorn wheat (*Triticum monococcum* L.; 2*n* = 2*x* = 14; A^m^A^m^) and domesticated emmer wheat (*Triticum turgidum* L.; 2*n* = 4*x* = 28; AABB) were disseminated across Asia, Europe and Africa as agriculture expanded. Then, about 8,000 years ago, a spontaneous hybridization of the wild diploid grass *Aegilops tauschii* Coss. (2*n* = 14; DD) with *T. turgidum* produced hexaploid common wheat (*T. aestivum*; 2*n* = 6*x* = 42; AABBDD). Such evolution of polyploid wheat varieties has been thoroughly described and discussed in a review by Matsuoka (2011).

Repetitive DNA comprises >80% of the genome of Triticeae plants (Smith and Flavell 1975). Bread wheat and barley possess genomes estimated to be 17 Gb and 5.1 Gb in size, respectively. The large size of their genomes and their rich repetitive sequences, as well as polyploidy in wheat, have made it challenging to understand fully the genomes of the Triticeae crops. However, there is a strong need for genomic resources to facilitate the discovery of new genes and develop genome-based breeding in wheat and barley because of the agricultural importance of these crops.

### Early sequence resource in Triticeae crops

**Fig. 1:**
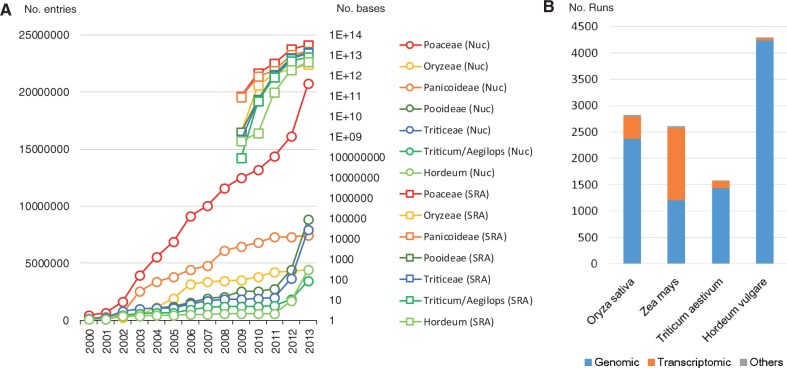
An overview of the publicly available sequence data in grasses. (A) The growth of sequence data in GenBank (number of entries) and the sequence read archive (number of bases). (B) Number of NGS runs of the four staple crops in Poaceae submitted to the sequence read archive (September 8, 2013).

In the past decade of the 21st century, the amount of sequence data from Poaceae species available in the public domain has dramatically increased (Fig. 1A). Since 2001, large-scale collection of cDNA and expressed sequence tag (EST) analysis using Sanger sequencing have been performed in several grass species, including wheat and barley (Mochida and Shinozaki 2010). Large-scale EST analysis has facilitated comprehensive acquisition of expressed genes as primary genomic information, and has also enabled monitoring of special and temporal gene expression and interspecific and intraspecific comparative analysis of expressed genes (Mochida et al. 2003, Ogihara et al. 2003, Zhang et al. 2004, Mochida et al. 2006). It has also facilitated the design of cDNA-oriented molecular markers and probes for oligo-microarrays (Kawaura et al. 2006, Torada et al. 2006, Mochida et al. 2008, Close et al. 2009, Sato and Takeda 2009, Sato et al. 2009a). Currently, there are >1.28 million EST entries in wheat and >0.5 million in barley in the dbEST of the National Center for Biotechnology Information (http://www.ncbi.nlm.nih.gov/dbEST/).

Full-length cDNA libraries and large-scale sequence data sets have played significant roles in various life science projects (Mochida and Shinozaki 2010). The sequence resources of full-length cDNA libraries can accurately identify transcribed regions and structural features (Ohyanagi et al. 2006, Sakai et al. 2013), such as transcription units, transcription start sites and transcriptional variants in completed or draft genome sequences. Full-length cDNA libraries have also contributed to functional analyses by enabling the creation of overexpression strains. The advent of function-based gene discovery via full-length cDNA overexpressor (FOX) gene hunting has enabled high-throughput discovery of functional genes associated with phenotypic changes (Kondou et al. 2009, Sakurai et al. 2011, Hong et al. 2013). Based on the biotinylated CAP trapper method (Carninci et al. 1996), large-scale analyses of full-length cDNAs have been conducted in wheat and barley (Kawaura et al. 2009, Sato et al. 2009b, Matsumoto et al. 2011). The TriFLDB database integrates full-length coding sequence information in wheat and barley (http://trifldb.psc.riken.jp). It provides functional annotation and associated information based on comparative analysis with other plant species (Mochida et al. 2009a).

Most of the contiguous genomic sequences identified in Triticeae before 2009 were obtained through map-based gene cloning or comparative studies. The chloroplast and mitochondrial genomes of hexaploid wheat were completely sequenced in 2002 and 2005, respectively (Ogihara et al. 2002, Ogihara et al. 2005). Knowledge of these whole-genome sequences of wheat organelles has enabled the investigation of evolution and RNA editing events of genes in the organelle genomes (Matsuoka et al. 2002, Yura et al. 2009). In addition to the Sanger-based sequence data, since 2009 NGS-based data of Pooideae plants have appeared in the Sequence Read Archive (SRA; http://www.ncbi.nlm.nih.gov/sra). The amount of deposited data from NGS has rapidly increased (Fig. 1A). So far, NGS technology has mainly been used to analyze genomic sequences of wheat and barley (Fig. 1B).

### Whole-genome analysis in Triticeae plants

*Barley* With the aim of constructing a high-quality reference sequence of the barley genome, an international consortium (International Barley Sequencing Consortium, http://barleygenome.org) was initiated in 2006 (Schulte et al. 2009). Barley genomic resources at that time were well reviewed by Sreenivasulu et al. (2008). The barley ESTs were applied to generate high-density genetic maps using innovative genotyping platforms (Stein et al. 2007, Close et al. 2009, Sato and Takeda 2009). High-density genetic maps, in turn, made it possible to compare the barley genome at the chromosome level with those of other genome-sequenced grasses (Mochida et al. 2008).

More recently, NGS technologies have been applied to accumulate information about the barley genome sequence (Wicker et al. 2006, Wicker et al. 2008, Wicker et al. 2009). An integrated approach called the genome zipper incorporates chromosome sorting, NGS technology, array hybridization and systematic exploitation of conserved synteny with model grasses and was used to build a virtual gene order of barley chromosome 1H (Mayer et al. 2009). It was possible to sort chromosome 1H by flow cytometric analysis because it was smaller than barley chromosomes 2H–7H. To sort those chromosomes, wheat–barley telosome addition lines (2HS–7HL arms) were used, and the genome zipper-based gene order in the whole-genome scale was represented in barley (Mayer et al. 2011) (http://mips.helmholtz-muenchen.de/plant/barley/gz/index.jsp).

The barley genome context was eventually published in the form of an integrated and ordered physical, genetic and functional sequence resource in 2012 (International Barley Genome Sequencing Consortium 2012). A 4.98 Gb physical map was constructed based on assembly of bacterial artificial chromosome (BAC) fingerprint contigs in the barley cultivar Morex, >3.9 Gb of which was anchored to an integrated genetic framework. Barley full-length cDNAs (Matsumoto et al. 2011) and RNA-seq data sets were used to annotate genes in the whole-genome shotgun assembly of barley. The result showed 79,379 transcript clusters, including 26,159 ‘high-confidence’ genes with homology support from other plant genes. The associated data set of the barley gene space is accessible from the MIPS barley genome database (http://mips.helmholtz-muenchen.de/plant/barley/). In the Ensembl plants, the whole-genome shotgun contigs anchored with FPC contigs in Morex barley have been displayed at genetic coordinates, which are approximations inferred from positional information in the barley physical maps (http://plants.ensembl.org/Hordeum_vulgare/Info/Index).

*Wheat* The International Wheat Genome Sequencing Consortium (www.wheatgenome.org) has used a chromosome by chromosome approach to mapping and sequencing 21 chromosomes of bread wheat, cultivar Chinese spring genome, using chromosome flow sorting. A physical map of wheat chromosome 3B was constructed as the first chromosome-scale physical map (Paux et al. 2008), which originated from sorted chromosome 3B (Safar et al. 2004). Double ditelosomic lines were used to construct chromosome arm-specific BAC libraries and physical contigs, for example, in chromosomes 1BL, 4A and 1A (Hernandez et al. 2012, Lucas et al. 2013, Philippe et al. 2013). Six arms of the group 1 chromosomes of bread wheat cv. Chinese spring, which were flow sorted, were sequenced by Roche/454 and compared with similar data sets from the homoeologous chromosome 1H of barley (Wicker et al. 2011). Using chromosome 3B, an improved version of the physical map was constructed and used to map about 3,000 transcripts (Rustenholz et al. 2011). Using chromosome sorting and NGS technologies, NGS-based survey sequencing analyses of each chromosome arm were performed. Data sets of NGS reads corresponding to chromosome arms are downloadable from SRA, such as ERP003210, ERP002108 and SRP007288. The online database WheatGenome.info (http://www.wheatgenome.info/) is providing an NGS-based data set derived from chromosome group 7 of wheat (Lai et al. 2012).

Brenchley et al. (2012) successfully deciphered the bread wheat genetic code using the whole-genome shotgun sequencing approach using 454 pyrosequencing and comparative analyses with diploid ancestral and progenitor genomes (Brenchley et al. 2012). In that study, Brenchley and colleagues identified around 96,000 genes and assigned two-thirds of them to the three component genomes (A, B and D) of hexaploid wheat. The associated data set of the wheat shotgun sequencing is available in the MIPS wheat genome database (http://mips.helmholtz-muenchen.de/plant/wheat/uk454survey/index.jsp).

Soon after the simultaneous publication of the compilation and analysis of the genome sequences of barley and wheat, two papers published in the same issue presented a draft genome sequences and analysis of two wheat progenitors, *Triticum urartu* and *A. tauschii* (Jia et al. 2013, Ling et al. 2013). To sequence the *T. urartu* genome, Ling and colleagues obtained 448.49 Gb of filtered sequence data and assembled them to scaffolds that represented 4.66 Gb, of which 66.9% comprised repetitive elements. They generated RNA-seq data from various organs and treatments and applied them to identify 34,879 protein-coding gene models (Ling et al. 2013). To sequence the *A. tauschii* genome, Jia et al. obtained an approximately 90-fold depth of short reads from libraries with various insert sizes and assembled them to scaffolds that represented 83.4% of the genome, of which 65.9% comprised transposable elements. They also generated RNA-seq data derived from various tissues and applied them to identify 43,150 protein-coding genes, of which 30,697 (71.1%) were anchored to chromosomes with an integrated high-density genetic map (Jia et al. 2013).

In summary, we have been able to access the deciphered genetic code of the four main Triticeae plants at the whole-genome scale (Table 1). The information from their genomes should be a primary resource for studies of their complex genomes and to make improvements in wheat and barley. Using these compilations of genomic information, genomic tools will be revitalized and new ones will be developed to accelerate the discovery of useful genes in wheat and barley. Genome-scale approaches could be applied further in Triticeae crops, coupled with NGS technologies, such as RNA-seq analysis to monitor transcriptomes with single-base resolution, chip-seq analysis to identify chromatin modifications or protein–DNA interactions, and exome sequencing to discover polymorphisms in genome scale (Mascher et al. 2013a).

**Table 1:** Sequence resources of four genome-sequenced plants in Triticeae

	*A. tauschii*	*T. urartu*	*T. aestivum*	*H. vulgare*
Whole-genome sequencing project	Jia et al. (2013)	Ling et al. (2013)	Brenchley et al. (2012)	International Barley Genome Sequencing Consortium (2012)
BioProject	PRJNA182898	PRJNA182347	PRJEB568	Listed in International Barley Genome Sequencing Consortium (2012) Supplemental Note 1
GenBank/EMBL/DDBJ	AOCO000000000	AOTI00000000	CALO00000000	
	AOCO010000000			
SRA	SRA030526	SRA030525	ERP000319	
	SRA062662	SRA066084		
	SRA063175	SRA064213		
No. of genomic scaffolds or contigs	429,891	499,221	945,079	6,437 physical map contigs
No. of protein sequences	33,849	24,169	94,000–96,000 (genes)	79,379 (transcript clusters)
Online resources	Ensembl Plants*^a^*	Ensembl Plants*^a^*	MIPS Wheat Genome Database*^c^*	MIPS barley genome database*^d^*
	gigadb*^b^*	gigadb*^b^*		Ensembl Plants*^a^*

*^a^* http://plants.ensembl.org/index.html

*^b^* http://gigadb.org/

*^c^* http://mips.helmholtz-muenchen.de/plant/wheat/uk454survey/index.jsp

*^d^* http://mips.helmholtz-muenchen.de/plant/barley/

### Other omics spectrums in Triticeae plants

*Transcriptomics* Comprehensive and high-throughput analysis of gene expression has become a significant approach for screening candidate genes, predicting gene function, discovering *cis*-regulatory motifs and characterizing transcriptional regulatory networks (Huang et al. 2011). A comprehensive collection of ESTs and full-length cDNA sequences was used to design probes of oligo-microarrays or GeneChips in model plants, as well as crops including wheat and barley. Rapid accumulation of large-scale gene expression data sets has provided us with an efficient and valuable resource for secondary uses, such as co-expression analyses (Hamada et al. 2011, Obayashi et al. 2011, Sasaki et al. 2011, Hirano et al. 2013a), and allow us to discover genes that are involved in certain metabolic pathways (Fukushima et al. 2013, Hirano et al. 2013b). In barley, a co-expression gene network was constructed using >1,000 sets of Affymetrix GeneChip data derived from 45 publicly available experimental series (Mochida et al. 2011a). Information resources providing co-expression gene networks of multiple plant species have been developed and are available on the web. PlaNet (http://aranet.mpimp-golm.mpg.de) provides information regarding co-expression gene networks constructed from microarray data sets of seven plant species, including wheat and barley (Mutwil et al. 2011). Microarray hybridization coupled with laser capture microdissection has been applied to monitor the transcriptome of specific cells or tissues of interest in plants (Takehisa et al. 2012, Kubo et al. 2013). In barley, laser capture microdissection microarray analysis was applied to analyze transcriptome in endosperm cells differentiate into transfer cells (Thiel et al. 2012).

Whole-transcriptome analysis using tiling array was an effective approach to explore novel transcription units, splicing variants and antisense transcripts, together with expression patterns (Matsui et al. 2008, Iida et al. 2011, Yoshimura et al. 2011, Endo et al. 2012). Because this method requires whole-genome information, it has been available only in a few genome-sequenced plants with small genomes. After NGS became available, RNA-seq rapidly became a popular approach to analyze the whole transcriptome in any species (Chang et al. 2011, Su et al. 2011, Swarbreck et al. 2011, Bai et al. 2013, Gil-Amado and Gomez-Jimenez 2013, Postnikova et al. 2013). The barley whole-genome sequencing project used 1.67 billion RNA-seq reads obtained from eight stages of barley development, and applied them to predict structures of transcription units (International Barley Genome Sequencing Consortium 2012). Dissecting the complex transcriptome in polyploidy species such as wheat in order to understand homoeolog-specific gene expression at the genome scale is challenging. Differential expression among homoeologs in the A, B and D genomes is often observed in the wheat transcriptome (Leaungthitikanchana et al. 2013, Mochida et al. 2003). RNA-seq analysis could be a promising approach to identify allele-specific and/or homoeolog-specific transcripts based on nucleotide polymorphisms in the sequenced read assembly. Recently, RNA-seq was applied to separate homoeologs in the tetraploid durum wheat transcriptome based on k-mer assembly strategies (Krasileva et al. 2013).

*Metabolomics* Over the last decade, metabolomics has become a powerful technique to identify metabolic features underlying complex phenotypes in biological systems (Okazaki and Saito 2012, Higashi and Saito 2013, Putri et al. 2013). Metabolome profiling provides a snapshot of the accumulation patterns of metabolites in response to various kinds of biological conditions, such as environments, developmental stages and genotypes. For example, metabolome profiling approaches have been used to evaluate stress responses in various plants, including wheat and barley (Widodo et al. 2009, Ishikawa et al. 2010, Bowne et al. 2012, Wu et al. 2013a). Recently, correlations between ionomic and metabolomic profiles in response to salt stress were investigated in wild and cultivated barley accessions (Wu et al. 2013b). Metabolome profiling has also been used to evaluate metabolic phenotypes of transgenic plants of wheat and barley (Baker et al. 2006, Kogel et al. 2010). Hormonomics is a specific, targeted use of metabolomics to analyze phytohormones. Simultaneous profiling of phytohormones and their derivatives (Kojima et al. 2009, Kanno et al. 2010) is a key approach to a holistic understanding of the plant hormone network associated with biological systems (Nomoto et al. 2012, Kamada-Nobusada et al. 2013).

The combined use of multiple omics technologies and integrated analysis of omics-based data sets have allowed us to study biological systems multilaterally. Integrated analysis of metabolome results and the microarray-based transcriptome have been widely applied to provide a multifaceted view of cellular systems in response to stress conditions, for example (Ishikawa et al. 2010, Watanabe et al. 2010, Yamakawa and Hakata 2010). More recently, the combined use of metabolome analysis and NGS-based transcriptome analysis has been an effective systems-based approach to explore gene regulatory networks superimposed with metabolic features of particular metabolic pathways (Yamazaki et al. 2013), and construct metabolic pathways (Van Moerkercke et al. 2013).

Recently, a number of information resources have been released or updated to provide tools for metabolome analysis. Recently published reviews have showcased information resources in plant metabolomics (Fukushima and Kusano 2013, Higashi and Saito 2013). In the following discussion, we will introduce two online tools for metabolomics and hormonomics that were recently released from RIKEN. PRIMe (http://prime.psc.riken.jp/), the Platform for RIKEN Metabolomics, is a website that was developed to assist systems approaches, ranging from metabolomics to transcriptomics. PRIMeLink, a newly designed tool in PRIMe, provides seamless and simultaneous access to the metabolome and transcriptome data sets (Sakurai et al. 2013). UniVIO (http://univio.psc.riken.jp/), the Uniformed Viewer for Integrative Omics, displays hormone–metabolome (hormonome) and transcriptome data in a single formatted heat map (Kudo et al. 2013).

Advanced applications of metabolomics technologies are providing new insight into molecular elucidation of plant functions. The use of metabolomics technologies combined with high-throughput and genome-scale genotyping technologies is allowing us to carry out genome-wide association studies of the accumulation patterns of metabolites in plants. These studies may provide promising links for narrowing the genotype–phenotype gap for complex agronomic traits (Riedelsheimer et al. 2012). Plant lipidomics is a new area in plant metabolomics focused on identifying novel and important roles played in plants by hydrophobic compounds (Okazaki et al. 2013). Metabolite imaging, a promising technology to image the distribution of metabolites among cells and tissues of plants, is expected to increase its power of resolution to provide unprecedented data on the distribution of metabolites at the subcellular level (Lee et al. 2012).

Changing the resolution and scale in genome-wide approaches, from single-cell resolution to life-scale and population-scale analyses, is expected to become an important solution to reveal biological systems in higher resolution. For single-cell or cell type-specific transcriptome analysis, linear amplification methods of RNAs or cDNAs that are compatible with NGS platforms have been developed and applied to various species (Osaka et al. 2013). Recently, cell type-specific metabolome analysis was demonstrated in Arabidopsis root (Moussaieff et al. 2013). Furthermore, thanks to an increase in throughput in analytical platforms, it has become possible to acquire omics profiles at a population scale and/or over time, throughout the life cycle of an organism. These advancements will significantly upgrade our molecular understanding of biological systems in plants and could be rapidly applied not only in model plants but also in economically important plants, including wheat and barley.

## Study of *Brachypodium* and its roles in Pooideae genomics

**Fig. 2:**
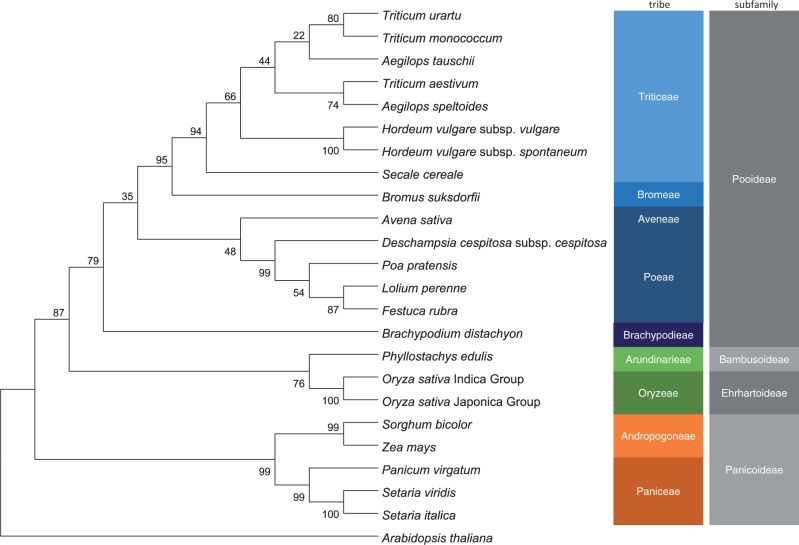
A phylogenetic tree of grass species based on the *ndhF* gene: YP 008239279 (*T. urartu*), YP 008239142 (*T. monococcum*), YP 008474350 (*A. tauschii*), AGP51333 (*T. aestivum*), YP 008474440 (*A. speltoides*), AGP50802 (*H. vulgare* subsp. *vulgare*), AGP50881 (*H. vulgare* subsp. *spontaneum*), YP 008239219 (*Secale cereale*), ABH02660 (*Bromus suksdorfii*), AAA64204 (*Avena sativa*), ABH02669 (*Deschampsia cespitosa* subsp. *cespitosa*), AAA64698 (*Poa pratensis*), YP 001531329 (*Lolium perenne*), ABH02677 (*Festuca rubra*), YP 002000532 (*B. distachyon*), YP 004733625 (*Phyllostachys edulis*), YP 654249 (*Oryza sativa* Indica group), NP 039441 (*O. sativa* Japonica group), YP 899454 (*Sorghum bicolor*), NP 043084 (*Zea mays*), YP 004841996 (*Panicum virgatum*), AAA64841 (*Setaria viridis*), AAM22087 (*S. italica*) and NP 051106 (*A. thaliana*). The phylogenetic tree was constructed with the aligned NdhF protein sequences by MEGA [version 5.2; http://www.megasoftware.net/ (Tamura et al. 2011)] using the Neighbor–Joining method with the following parameters: Poisson model, complete deletion and bootstrap method. The bootstrap values from 1,000 replicates are given at each node. The NdhF protein sequences were aligned by ClustalW implemented in MEGA.

*Arabidopsis thaliana* (L.) Heynh has been used as the model organism in plant biology for almost 30 years. The success of Arabidopsis research provides proof of the importance of a model organism. *Brachypodium distachyon* was proposed as a model system by Draper et al. in 2001, for analyzing genetic functions and biological systems in temperate grasses, cool-season cereals and dedicated biofuel crops (Draper et al. 2001, Mur et al. 2011). The species has tractable features, a short life cycle, small plant size, facile transformation, simple growth requirements and a small genome. It belongs to the Pooideae subfamily (Fig. 2) and serves as a model system for major crops, such as wheat, barley, rye and oats (Bevan et al. 2010). In 2010, the whole-genome sequence of the inbred line Bd21 was published, making it the first completely genome-sequenced species of the Pooideae subfamily (International Brachypodium Initiative 2010).

### *Brachypodium* resources

*Brachypodium* has garnered attention as a model system, and a number of projects to develop genomic resources have been initiated at various institutions. Already, several reviews have described available resources related to *Brachypodium* (Brkljacic et al. 2011, Mur et al. 2011). Here, we focused on recently published *Brachypodium* resources (Table 2).

**Table 2:** Online resources for *Brachypodium* research

	Description	URLs
Brachypodium.org	Genome sequence	http://brachypodium.org/
	Gene annotation	
	ESTs	
	RNA-seq data sets	
	Resequencing data sets	
Phytozome	Genome sequence	http://www.phytozome.net/
	Gene annotation	
Brachypodium genome database–MIPS	Genome sequence	http://mips.helmholtz-muenchen.de/plant/brachypodium/
	Gene annotation	
	Protein doman	
	Comparative genome view	
NCBI RefSeq	Gene annotation as RefSeq entry	http://www.ncbi.nlm.nih.gov/refseq/
Ensembl Plants	Genome sequence	http://plants.ensembl.org/Brachypodium_distachyon/Info/Index
	Gene annotation	
	Wheat transcriptome alignments	
Gramene	Gene annotation	http://www.gramene.org/
	Comparative maps	
	Pathways (BrachyCyc)	
RIKEN Brachypodium Full-length cDNA Database	Gene annotation	http://brachy.bmep.riken.jp/ver.1/index.pl
	Full-length cDNAs	
	Triticeae transcriptome alignment	
PlaNet	Gene expression profiles based on the GeneChip	http://aranet.mpimp-golm.mpg.de/
	Co-expression analysis	
BrachyTAG	T-DNA tag lines	http://www.brachytag.org/
	FTS on the Bd21 genome	
Western Regional Research Center *Brachypodium*	T-DNA tag lines	http://brachypodium.pw.usda.gov/
	FTS on the Bd21 genome	
BRACHYTIL	TILLING lines	http://urgv.evry.inra.fr/UTILLdb
GramineaeTFDB	Transcription factors	http://gramineaetfdb.psc.riken.jp/
PlantTFDB	Transcription factors	
BrachyCyc	Pathways	http://pathway.gramene.org/BRACHY/class-tree?object=Pathways
	Gene Ontology	
KEGG	Pathways	http://www.genome.jp/kegg/
	Gene annotation	
Mapman	Mapping to MapMan Ontology	http://mapman.gabipd.org/
Plant Genome Duplication Database	Syntenic relationships	http://chibba.agtec.uga.edu/duplication/
PLAZA	Syntenic relationships	http://bioinformatics.psb.ugent.be/plaza/
	Gene annotation	
	Gene Ontology	
E-TALEN	Web service to design TALENs	http://www.e-talen.org/E-TALEN/

*Genome sequence and annotations* The current version of the reference genome sequence of Bd21 is the Joint Genome Institute v1.0 8x assembly with approximately 272 Mb and a 99.6% deciphered genome, arranged in five chromosomes with 78 unmapped scaffolds (International Brachypodium Initiative 2010). The sequence data set of the Bd21 genome is available from a genomic portal, Brachypodium.org, as well as from Phytozome (http://www.phytozome.net/brachy.php), MIPS (http://mips.helmholtz-muenchen.de/plant/brachypodium/) and Ensembl Plants (http://plants.ensembl.org/Brachypodium_distachyon/Info/Index). The high-quality reference genome sequence of Bd21 could be an essential resource for every genome-oriented application using *Brachypodium*. The representative gene structural annotations of Bd21 are available from Phytozome and from MIPS, and each contains the same number of annotated gene models on identical loci, with slightly different annotations, mainly in the untranslated regions. More recently, we updated gene structure annotations of the Bd21 genome by comparing full-length cDNA sequences with the publicly available annotations from the Phytozome and MIPS. About 10,000 non-redundant gene models were supported by the full-length cDNAs and about 6,000 showed some modifications in the gene model. We also found about 580 novel gene models, including 362 newly identified transcription units (Mochida et al. 2013a).

Whole-genome re-sequencing data have recently appeared in the public domain, representing the progress of the re-sequencing project in the Joint Genome Institute-Department of Energy. In the Brachypodium.org data set, Bd21-3, Bd3-1, Bd30-1, Bd1-1, Koz-3 and BdTR12C are currently available with analyzed data sets. In addition to those, the row data sets of 20 other accessions, BdTR8i, Mur1, Foz1, Mig3, ABR5, Mon3, ABR9, ABR2, BdTR13a, BdTR7a, BdTR1i, Tek-2, Koz-1, Gaz-8, BdTR2G, BdTR10C, Adi-10, Arn1, ABR6 and ABR9, released from JGI, have been deposited in the public sequence read archives (September 24, 2013).

*Transcriptome* The National Center for Biotechnology Information database dbEST contains 128,092 Sanger ESTs from *B. distachyon*. Brachypodium.org provides EST data sets from Bd21 and some other accessions (ftp://brachypodium.org/brachypodium.org/ESTs/). The data set of the ‘454 sequencing of Brachypodium distachyon EST project’ are available from the National Center for Biotechnology Information’s SRA (SRX014502–SRX014507). We recently constructed a mixed full-length cDNA library from 21 different tissues of Bd21 and obtained 78,163 ESTs from both the 5′ and 3′ ends of about 40,000 clones, which were applied to update structural annotations, as described above. We integrated the *Brachypodium* full-length cDNAs and updated gene structures with available sequence resources in wheat and barley in a web-accessible database, the RIKEN *Brachypodium* FL cDNA database (RBFLDB, http://brachy.bmep.riken.jp/ver.1/index.pl).

The Affimetrix GeneChip is a combination of genome tiling and exon scanning designed for multiple uses, such as expression profiling and ChIP-chip studies. Information related to the probe design of the GeneChip is available from Brachypodium.org (ftp://brachypodium.org/brachypodium.org/Affymetrix/Current/). Expression profiles of *Brachypodium* genes in 36 tissues or conditions, and co-expression networks, which are based on hybridization experiments using the GeneChip, are available from the PlaNet website http://aranet.mpimp-golm.mpg.de/. RNA-seq transcriptome data based on Illumina sequencing were generated to annotate gene structure for the sequencing project of Bd21, available from the National Center for Biotechnology Information’s SRA (SRA010177) and from Brachypodium.org (ftp://brachypodium.org/brachypodium.org/Bd21_Original_RNaseq/) as the Bd21 original RNA-seq data set. More recently, RNA-seq analysis was performed to compare the transcriptome in species representing three Poaceae subgroups, i.e. the Pooideae (*B. distachyon*), the Panicoideae (sorghum) and the Ehrhartoideae (rice) (Davidson et al. 2012).

*Genetic resources* Comprehensive collections of mutant lines are essential bioresources to promote forward and reverse genetics. Using the efficient *Agrobacterium tumefaciens*-mediated transformation method, a project to create a *Brachypodium* T-DNA collection was initiated. The BrachyTAG collection (http://www.brachytag.org) and the Western Regional Research Center *Brachypodium* insertional mutant population (http://brachypodium.pw.usda.gov) are available from searchable websites, providing *Brachypodium* T-DNA lines with flanking sequence tags (Thole et al. 2010, Bragg et al. 2012). TILLING (Targeting Induced Local Lesions In Genomes), a useful platform to identify an allelic series of mutants, has been widely developed in model organisms and crops (Saito et al. 2011, Wang et al. 2012). A TILLING platform called BRACHYTIL, created for the inbred line Bd21-3 mutagenized by NaN_3_, was released, and phenotypes were organized in a phenotypic tree available at http://urgv.evry.inra.fr/UTILLdb (Dalmais et al. 2013).

*Brachypodium* genetic linkage maps serve as important tools for a range of molecular genetic analyses. A genetic linkage map was developed with an F_2_ population from a cross between the lines Bd3-1 and Bd21 using simple sequence repeat (SSR)-based markers (Garvin et al. 2010). A genetic linkage map of an F_2_ mapping population of 476 individuals from the same cross combination was generated using single nucleotide polymorphism (SNP) markers with the GoldenGate assay (Huo et al. 2011). Then an F(6:7) recombinant inbred line population from the same cross combination was generated and used to conduct fine mapping of the *Bsr1, Barley stripe mosaic virus* (BSMV) resistance gene (Cui et al. 2012).

Site-specific nuclease technologies, zinc finger nucleases, transcription activator-like effector nucleases (TALENs) and clustered regulatory interspaced short palindromic repeat/Cas-based RNA-guided DNA endonucleases are innovative tools for genome engineering (Gaj et al. 2013). Recently, efficient genome modification using the TALEN method was demonstrated at eight loci in *Brachypodium* (Shan et al. 2013). The latest update of E-TALEN (http://www.e-talen.org/), which is a tool to design and evaluate TALEN, supported *Brachypodium* as one of the additional organisms (Heigwer et al. 2013).

### Integration of genomic resources in Pooideae

The recent progress in genomic resources and analytical tools for use in Triticeae crops and *Brachypodium* facilitates gene discovery studies and functional analysis of genes involved in specific properties of Pooideae grass species. Comparative genomics and integration of available genomic instances in the Pooideae species should synergistically enable us to use genomic features to accelerate the gene discovery process, as well as complement and enrich our knowledge of structures and functions of orthologous genes in this subfamily.

*Comparative genomics in Pooideae* Conserved synteny is an important genomic property in genome evolution and is helpful in molecular marker development and positional gene cloning. Synteny among genome-sequenced Poaceae species, including rice, sorghum and *Brachypodium*, was used to develop a virtual gene order map (‘genome zipper’) for barley (*H. vulgare*), as mentioned earlier. The genome zipper approach has now become a key genomic technology to unlock complex genomes in the Pooideae subfamily (Spannagl et al. 2013), and its use has been applied in other Pooideae species, such as in wheat chromosome 4A (Hernandez et al. 2012), rye chromosome B (Martis et al. 2012) and *Lolium* (Pfeifer et al. 2013). The synteny between *Brachypodium* and Triticeae crops has assisted scientists in narrowing down candidate causal genes of mutants and quantitative trait loci (QTLs) in wheat and barley (Griffiths et al. 2006, Mueller et al. 2012, Liu et al. 2013). In the whole-genome shotgun sequencing of bread wheat, the wheat 454 reads, SNPs and genetic maps of wheat were anchored to the *Brachypodium* genome (Brenchley et al. 2012). Comparative use of the high-quality genome sequence of *Brachypodium* Bd21 has helped us to reconstruct a syntenically ordered draft genome in Pooideae species. These examples of comparative analysis in Pooideae show the significance of integrating genomic instances of Triticeae crops with those in *Brachypodium*.

**Fig. 3:**
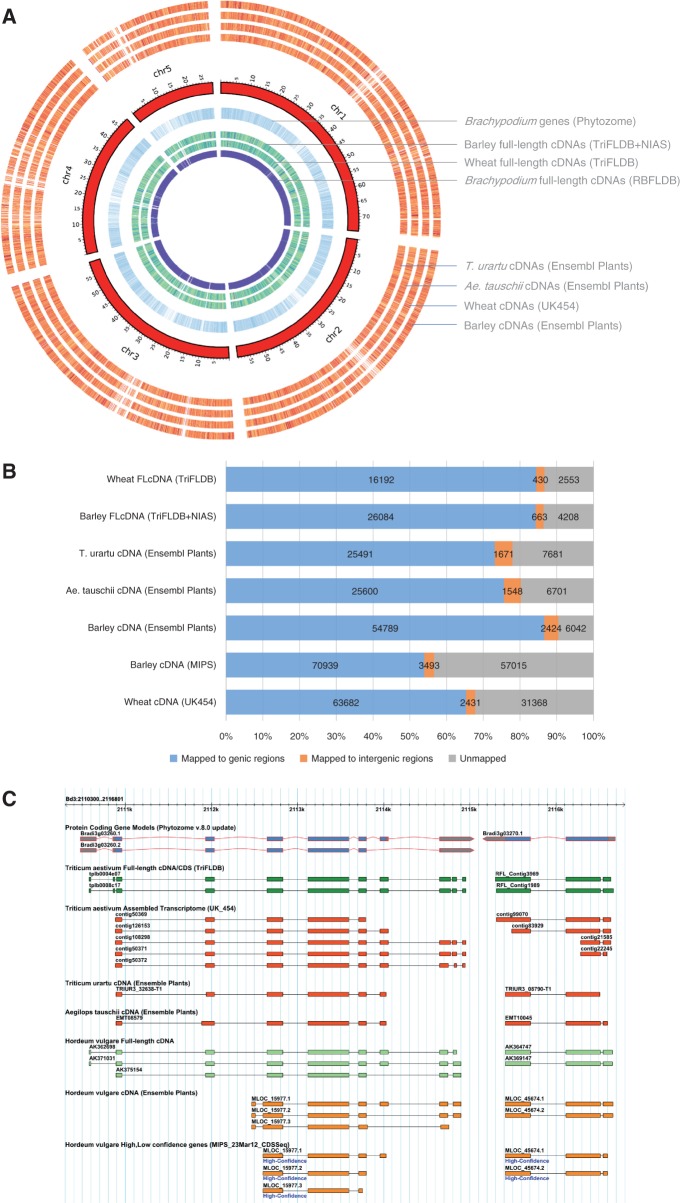
Comparative mapping of Triticeae cDNAs to the *Brachypodium* genome. A data set of full-length cDNAs of barley (GenBank accession Nos. AK353559–AK377172 deposited by NIAS, and available in TriFLDB) and wheat retrieved from TriFLDB, shotgun genome analysis of wheat by Roche 454 sequencing (downloaded from MIPS), cDNAs of *T. urartu* and *A. taucschii* (downloaded from Ensembl Plants) and barley cDNAs annotated on the cv. Morex genome (downloaded from MIPS and from Ensembl Plants) were used to the comparative mapping analysis. Cross-species mapping using cDNA data sets for querying the *Brachypodium* Bd21 genome was performed by sim4 with default parameter settings, followed by a BLASTN search, with e-value cut-offs of <1e-20. (A) An overview of the mapping results represented by a circular image. (B) Numbers of queries of wheat full-length cDNAs [Wheat FLcDNA (TriFLDB) and those of barley (Barley FLcDNA (TriFLDB + NIAS)], cDNAs annotated in the draft sequences of *T. urartu* [T. urartu cDNA (Ensembl Plants)] and of *A. tauschii* [Ae. tauschii cDNA (Ensembl Plants)], gene models annotated in the barley Morex genome of MIPS [Barley cDNA (MIPS)] and those of Ensembl Plants [Barley cDNA (Ensembl Plants)] and wheat cDNAs from the shotgun genome analysis [Wheat cDNA (UK454)], respectively, mapped to the genic or intergenic regions of the *Brachypodium* genome, or not mapped, are represented. (C) An example screen shot of a genome browser for mapped cDNAs to the *Brachypodium* genome aligned with annotated genes of *Brachypodium*. The mapping results were implemented in the Gbrowse interface of the RBFLDB web site and are available from http://brachy.bmep.riken.jp/ver.1/tools.pl?t=gbrowse&sp=Bdi.

Comparative mapping analysis to identify closest counterparts is one of the practical approaches to achieve significant data integration of sequence resources in Pooideae. We performed a comparative mapping analysis of *Brachypodium*, wheat and barley, based on cDNAs and genomic assemblies. To establish the inter-relationships between Pooideae transcripts and genomic sequences, comparative mapping analysis was performed using available comprehensive cDNA sequences from each species of wheat, barley and *Brachypodium* as queries against the genomic sequences of *Brachypodium* and barley. We demonstrated that the data sets allowed us immediately to identify full-length cDNAs of putative orthologous genes in counter-species of Pooideae, and to compare gene structures conserved among putative orthologs (Mochida et al. 2013a). As a further extension, we also mapped transcript sequences of annotated genes in *A. tauschii* and *T. urartu* genes to the *Brachypodium* genome. Fig. 3A represents an overall image of the mapping results of cDNAs of wheat, barley and *Brachypodium*, as well as annotated gene models of barley, *A. tauschii* and *T. urartu* with annotated *Brachypodium* genes located on its five chromosomes. Most of the queried transcripts were allocated to the annotated gene regions of the *Brachypodium* genome; some were allocated to the non-annotated regions. These results suggest that there are potential transcription units of *Brachypodium* with conserved homology to cDNAs in Triticeae plants that have not yet been annotated (Mochida et al. 2013a) (Fig. 3B). The *Brachypodium* genome browser in RBFLDB (http://brachy.bmep.riken.jp/ver.1/tools.pl?t=gbrowse&sp=Bdi) consists of annotation tracks for the results of the comparative mapping, which should help to confirm conserved gene structures among orthologs in the Pooideae species (Fig. 3C). The integration of Triticeae sequence resources with the *Brachypodium* genome should be useful for annotating the genomes of the Pooideae, performing comparative studies and yielding putative orthologs among Pooideae species.

The identification of orthologous groups is another practical approach to integrate genomic knowledge among related species. This work encompasses useful genome annotation, studies of gene/protein evolution and the identification of taxonomically restricted sequences (Li et al. 2003). Orthologous identification using OrthoMCL has been widely used to determine the distribution of orthologous gene families in genome-sequenced species compared with other species. The *Brachypodium* proteome data set was analyzed using OrthoMCL to detect *Brachypodium*- and Pooideae-specific genes (International Brachypodium Initiative 2010). Proteome data sets of *A. tauschii* and *T. urartu* were used, respectively, to analyze the distribution of orthologous gene families in the Poaceae family, based on the OrthoMCL analysis of *Brachypodium*, sorghum, rice and barley, and of *Brachypodium*, sorghum, rice and maize (Jia et al. 2013, Ling et al. 2013). Genome-wide assignments of protein-coding genes to homologous gene families provide us with a foundation to perform subsequent evolutionary studies for functional predication of genes.

After completing genome sequencing and full-length cDNA projects in human, the mouse and rice, ‘jamboree-style’ annotation meetings were orchestrated, such as H-inv (the Human Invitational Annotation project) (Imanishi et al. 2004), FANTOM (Functional Annotation Of Mouse) (Maeda et al. 2006) and RAP (Rice Annotation Projects) (Ohyanagi et al. 2006, Sakai et al. 2013). Accurate gene annotations of these species have played essential roles in their various post-genome projects, and associated databases have become hubs of information resources for each species. In the Pooideae subfamily, a high-quality genome sequence of *Brachypodium* Bd21 and enriched gene annotations will provide a firm foothold in comparative genomics with Triticeae crops. Integrating and cross-referencing information resources between *Brachypodium* and Triticeae plants could improve gene annotation work and allow researchers to infer shared orthologs and specific or divergent genes in this subfamily.

Some function-related annotations of genes, such as ontology, pathways and conserved sequence features, also play important roles in the prediction of biological functions of genes, as well as allowing us to grasp an overview of genome-scale instances. The Gene Ontology (GO) project has been widely used for functional classification of genes in various situations. Mapping results of *Brachypodium* genes to the GO terms are available in some online resources, Plaza (ftp://ftp.psb.ugent.be/pub/plaza/plaza_public_02_5/GO/go.bdi.csv.gz) and RBFLDB (http://brachy.bmep.riken.jp/ver.1/static/download/iprscan.results.tab.txt.gz). Regarding metabolic pathways, BrachyCyc version 2.0 is available from the Gramene website (http://pathway.gramene.org/gramene/brachycyc.shtml). With the availability of complete genome sequences, we have been able to compile catalogs describing the function and organization of regulatory systems of transcription factors (TFs) and further integrate information on TFs with knowledge related to their functions (Mochida et al. 2009b, Mochida et al. 2010, Mochida et al. 2013b, Naika et al. 2013). PlantTFDB (http://planttfdb_v2.cbi.edu.cn/index.php?sp=Bdi) and GramineaeTFDB (http://gramineaetfdb.psc.riken.jp/) provide information about genes encoding TFs in the *Brachypodium* genome (Mochida et al. 2011b, Zhang et al. 2011). Information about putative *cis*-elements located in the promoter regions (–3,000 bp from the TU start) in the *Brachypodium* genome is available from RBFLDB (http://brachy.bmep.riken.jp/ver.1/dl.pl). These resources could function as contextual filters for meta-analysis in omics-based approaches to narrow down candidate genes and build hypotheses for further analysis in *Brachypodium* and in Triticeae plants.

### *Brachypodium* to Triticeae crops

Comparative genomics studies among Poaceae species have suggested that *Brachypodium* is a better, more closely related model than rice for Triticeae crops. *Brachypodium* possesses all the features of a modern model organism. It is therefore advantageous to accelerate gene discovery and translational research to improve the Triticeae crops. Recently, *Brachypodium* has become an accepted model plant for research related to various physiological phenomena, particularly those observed in monocots; many citations demonstrate the utility of *Brachypodium* as a model system. Here, we introduce several typical examples recently studied using *Brachypodium*, which could provide a useful model system for better understanding of the biological systems and transferring knowledge to Triticeae crops.

Triticeae crops suffer severe yield losses from biotic stresses such as pathogens and pests. For example, viruses such as BSMV and the *Wheat streak mosaic virus*, which infect the Triticeae crops, have been studied using dicot host plants to dissect viral processes and determine how the pathogens cause disease. However, the underlying mechanisms of immunity remain largely unknown, especially the molecular basis of disease in monocot plants. Pathogens infecting *B. distachyon* and *Brachypodium sylvaticum* were listed by Mandadi and Scholthof (2013), and the reported bacterial, fungal and viral pathogens that infect *Brachypodium* species will assist further studies in better understanding the molecular basis of disease in monocots (Mandadi and Scholthof 2013). For example, to analyze the interaction of the *Panicum mosaic virus* (PMV) and its satellite virus (SPMV) with host plants, Mandadi and Scholthof investigated changes in the transcriptome of control *Brachypodium* plants and those infected by both viruses, using the custom-designed Affymetrix GeneChip (Mandadi and Scholthof 2012). More recently, the *Puccinia graminis–Brachypodium* pathosystem was examined for its potential to demonstrate incompatibility and non-host resistance in *P. graminis* using eight inbred *Brachypodium* lines (Figueroa et al. 2013). The *Fusarium* pathogen is the causal agent of Fusarium head blight (FHB) of small grain cereals including wheat and barley, which is a global problem due to the reduced yield and quality as well as to the contamination of mycotoxins. Peraldi et al (2011) showed disease symptoms on *Brachypodium* spikes and the accumulation of a mycotoxin within floral tissues, which were highly similar to those in wheat (Peraldi et al. 2011). Hence, as the number of Triticeae crop pathogens that also can infect *Brachypodium* species increases, *Brachypodium* will provide a significant model system to elucidate the molecular basis of interactions with those pathogens and will accelerate translational studies to wheat and barley.

For temperate grass species such as wheat and barley, *Brachypodium* provides a good model system to investigate the responses of biological systems to temperature stresses. Li et al. (2012) examined potential uses of *Brachypodium* as a model for cold stress responses in the Pooideae subfamily. They described the significance of using *Brachypodium* in studies of cold stress responses in Pooideae based on comparative genomics. They also noted that the evolutionary history of key genes for low temperature responses differed among *Brachypodium* and core Pooideae species (Li et al. 2012). Recently, the thermal response of *Brachypodium* was analyzed based on transcriptomics using the Affymetrix GeneChip, chromatin modification with Chromatin-IP analysis and molecular genetics with RNA interference (RNAi). In this study, the authors indicated that H2A.Z-nucleosomes are important to coordinate sensitivity to increased temperature during grain development in *Brachypodium* (Boden et al. 2013). Plant response to drought has been extensively studied in Arabidopsis and in a few grass species (Hirayama and Shinozaki 2010). *Brachypodium* may possess specific attributes to adapt to or tolerate drought because of its geographic origin, and these attributes may be translated to related crop species, such as wheat and barley. Transcriptome profiles using the Affymetrix GeneChip of different developmental leaf zones illustrated different responses to drought in *Brachypodium* (Verelst et al. 2013).These examples represent the usefulness of *Brachypodium* as a model system in abiotic stress studies of temperate grass species with critical genomic tools and tractable biological features. In previous studies, the function of genes in wheat and barley has often been studied in transgenic plants such as Arabidopsis, tobacco and rice (Brini et al. 2011, Hu et al. 2012). However, the physiologically similar properties of *Brachypodium* and Triticeae crops provide opportunities to promote the functional characterization of genes in wheat and barley, including those involved in abiotic stress responses and tolerance of harsh conditions.

*Brachypodium* is a promising model system in some additional areas. It has already been widely used in studies of cellulosic biomass production and cell wall biosynthesis (Rancour et al. 2012). *Brachypodium* has provided a model system to understand biogenesis of type II cell wall in grasses, a type of cell wall that is structurally different from type I in dicots (Yokoyama and Nishitani 2004). Based on a phylogenetic and expression analysis of genes encoding Yellow Stripe-Like (YSL) transporters, *Brachypodium* was also proposed as a promising species for studies of iron homeostasis in grasses (Yordem et al. 2011). Recently, a similarity search in a barley EST database, using HvYS1 as the query in barley, identified HvYSL5 and HvYSL2 (Araki et al. 2011, Zheng et al. 2011). The *Brachypodium* orthologs of the YSL family offer a model system in comparative genomics of Fe distribution and translocation in grasses. Furthermore, in a recent review article by Chochois et al. (2012), the authors proposed *Brachypodium* as a model system for root research and to accelerate the identification of genes for wheat root improvement. *Brachypodium* and wheat have very similar root anatomies, distinct from the roots of rice, which are specialized to overcome anaerobic conditions associated with submerged roots (Chochois et al. 2012).

## Future prospects for crop improvements

As climate change progresses, creating varieties that can adapt to environmental stresses is one of the most important targets of crop breeding. Although our understanding of plant systems’ responses to environmental stresses have greatly increased in the last decade, applying the knowledge from basic research to improve the environmental stress tolerance of crops will be a challenge (Qin et al. 2011). Recently, a number of genes involved in responses to and tolerance of environmental stresses were identified and characterized in wheat and barley (Conde et al. 2011, Horie et al. 2011). Rapid advances in genomic tools with NGS applications in Triticeae and in *Brachypodium* will definitely accelerate gene discovery research in Triticeae crops. In addition to the sequence resources at the whole-genome scale, some important areas for crop improvement are being advanced by new experimental and computational approaches that use omics and mathematics.

One of the important areas is plant epigenetics, which has recently gained attention as a genomic regulatory system, not only for developmental controls but also for responses to environmental stresses and as a possible new source of beneficial traits for plant breeding (Mirouze and Paszkowski 2011, Kim et al. 2012b, Kinoshita and Jacobsen 2012). For example, histone modifications were shown to be correlated with the inactivation of drought-inducible genes, such as *RD20*, *RD29A* and *AtGOLS2*, during recovery by rehydration in Arabidopsis (Kim et al. 2012a). The epigenomics approach, involving whole-genome-scale epigenetics analysis, should elucidate genome-wide chromatin regulation in abiotic stress responses (Ikeda 2012). The data set of whole-genome sequences of Triticeae plants, at least for the gene space of their genomes, will provide opportunities to study epigenomic phenomena in wheat and barley and will potentially allow epigenetic knowledge to be applied to their improvements in the near future.

Another important area is mining alleles for beneficial traits in plants. Whole-genome reference sequences and platforms for high-throughput genotyping are providing opportunities to improve our understanding of the history of plant domestication and to apply evolutionary knowledge of the crop genome to crop improvements (Morrell et al. 2011). Chip-based and NGS-based platforms for whole-genome-scale genotyping are available for a number of genome-sequenced organisms (Chen et al. 2013) and also for wheat and barley (Sato et al. 2011, Cavanagh et al. 2013). High-throughput genotyping with dense genome-wide markers has allowed us to identify genomic regions associated with complex agronomic traits. Genome-wide association studies have been performed not only in Arabidopsis but also in various crops, including wheat and barley, using NGS-based whole-genome re-sequencing or microarray-based SNP genotyping (Huang et al. 2010, Pasam et al. 2012, Cavanagh et al. 2013, Morris et al. 2013). For species with large complex genomes, such as barley and wheat, reduced genomic representation using restriction enzymes has been an efficient approach, coupled with NGS-based DNA polymorphism discovery. Genotyping-by-sequencing (GBS) and restriction-associated DNA sequencing (RAD-seq) were developed and rapidly popularized for whole-genome profiling of DNA polymorphisms in many species, including barley and wheat (Poland et al. 2012, Mascher et al. 2013b).

The application of NGS technology to next-generation populations could provide an efficient strategy to identify loci that contribute to a complex trait. The nested association mapping (NAM) population, which is based on crossing diverse strains to a reference parent, has successfully been applied to a number of traits in maize (Kump et al. 2011, Tian et al. 2011). The multiparent advanced generation intercross (MAGIC) population or the recombinant inbred advanced intercross line (RIAIL) population also will help improve genetic mapping and advance breeding programs of crops, including barley and wheat (Cavanagh et al. 2008, Kover et al. 2009, Huang et al. 2012).

Based on the evolutionary history of a target crop, the discovery of loci responsible for adaptive evolution has been a promising approach to identify loci contributing to adaptation in the agronomic environment. Geographic distribution of the target locus represents patterns of historical selection of alleles that are adaptive to a local agronomic environment (Saisho et al. 2011). The geographic distribution of genotypes superimposed on information about local environmental conditions may inspire us to discover allelic combinations that would be of value in forecasted environments. Furthermore, the integration of genotype and epigenomic profiles of each strain (epigenotype) will provide important genomic information for dissecting the contribution of epigenetic and genetic variations to phenotypic changes in responses to the environment (Schmitz et al. 2013).

Crops in the field are continuously exposed to various environmental signals and must respond to fluctuating conditions. In order to translate knowledge more effectively from laboratories to field conditions in agriculture, we must understand the omics dynamics of crops in response to endogenous and exogenous perturbations under fluctuating field conditions. Recent advances in high-throughput technology allow us to measure changes in the omics of plants, not only under controlled conditions but also under natural conditions (Izawa et al. 2011, Richards et al. 2012, Dal Santo et al. 2013). In addition to the omics analysis in natural environments, to gain a mechanistic understanding of biological systems, mathematical modeling and simulation approaches are increasingly used in studies of plants’ cellular metabolism, growth, developmental processes and responses to the environment (Aikawa et al. 2010, De Vos et al. 2012, Katsuragi et al. 2013). In rice, a successful model and prediction of genome-wide transcriptional changes under fluctuating field conditions was recently represented based on transcriptome data and the corresponding meteorological data (Nagano et al. 2012). As we work to improve crops, we must take ongoing climate change into consideration. The omics-based data set of plants in natural environments may be a substantial resource to generate models for predicting how a plant system will react under field conditions it has not experienced, such as warmer, more rainy or less rainy. The combination of such a systems-based approach and genome-wide evolutionary analyses to identify beneficial genes will provide an opportunity to apply our wisdom of the past to crop breeding for the future. It may be a pre-emptive strategy to ensure sustainable food production under the changed environments of the future.

## Conclusions

Available tools for Triticeae genomics have dramatically advanced in recent years. Rapidly increasing sequence data sets and associated resources for Pooideae will potentially accelerate the gene discovery process and improve our understanding of their biological functions. We urgently need to integrate fragmented knowledge and translate genome-based wisdom to achieve practical improvements in the Triticeae crops. Integration of genomic information and resources from the model grass *Brachypodium* and those from Triticeae crops should make a huge contribution to gene discovery and improved understanding of gene functions, as well as transferring knowledge to the Triticeae crops. Recent advanced analytical platforms and applications based on NGS technologies have provided opportunities to advance radically the discovery of genes and alleles for beneficial traits despite the genome complexity of Triticeae species. The promising combination of these approaches with systems approaches and mathematical modeling will help us achieve crop improvements to ensure a sustainable agriculture in the future.

## Funding

This work was supported by a grant from a strategic research project in RIKEN Biomass engineering program.

## Acknowledgments

The authors thank Tetsuya Sakurai, Takuhiro Yoshida of the RIKEN Center for Sustainable Resource Science for their data generation of cDNA mapping among Pooideae plants. The authors also thank Daisuke Saisho of Okayama University for his valuable comments and discussions on this manuscript.

## Disclosures

The authors have no conflicts of interest to declare.

## Abbreviations

BAC: bacterial artificial chromosome
BSMV: *Barley stripe mosaic virus*
EST: expressed sequence tag
FOX: full-length cDNA overexpressor
GO: Gene Ontology
NGS: next-generation sequencing
QTL: quantitative trait locus
SNP: single nucleotide polymorphism
SRA: sequence read archive
TF: transcription factor
TILLING: Targeting Induced Local Lesions IN Genomes
TALEN: transcription activator-like effector nuclease
